# Tracking the emergence of synthetic biology

**DOI:** 10.1007/s11192-017-2452-5

**Published:** 2017-07-01

**Authors:** Philip Shapira, Seokbeom Kwon, Jan Youtie

**Affiliations:** 1 Manchester Institute of Innovation Research, Alliance Manchester Business School, University of Manchester, Manchester, M13 9PL UK; 2 School of Public Policy, Georgia Institute of Technology, Atlanta, GA 30332-0345 USA; 3 Manchester Synthetic Biology Research Centre for Fine and Speciality Chemicals, Manchester Institute of Biotechnology, University of Manchester, Manchester, M1 7DN UK; 4 Enterprise Innovation Institute, Georgia Institute of Technology, Atlanta, GA 30308 USA

**Keywords:** Emerging technology, Synthetic biology, Bibliometric analysis, Search strategy, Map of science, Research sponsors, I23, O31, 032, 038

## Abstract

Synthetic biology is an emerging domain that combines biological and engineering concepts and which has seen rapid growth in research, innovation, and policy interest in recent years. This paper contributes to efforts to delineate this emerging domain by presenting a newly constructed bibliometric definition of synthetic biology. Our approach is dimensioned from a core set of papers in synthetic biology, using procedures to obtain benchmark synthetic biology publication records, extract keywords from these benchmark records, and refine the keywords, supplemented with articles published in dedicated synthetic biology journals. We compare our search strategy with other recent bibliometric approaches to define synthetic biology, using a common source of publication data for the period from 2000 to 2015. The paper details the rapid growth and international spread of research in synthetic biology in recent years, demonstrates that diverse research disciplines are contributing to the multidisciplinary development of synthetic biology research, and visualizes this by profiling synthetic biology research on the map of science. We further show the roles of a relatively concentrated set of research sponsors in funding the growth and trajectories of synthetic biology. In addition to discussing these analyses, the paper notes limitations and suggests lines for further work.

## Introduction

Synthetic biology is, according to a National Academy of Sciences (2013, 2) report of working parties from the US, the UK, and China, “an emerging discipline that combines both scientific and engineering approaches to the study and manipulation of biology.” Similar descriptions have been put forward by other commissions and studies. For example, a joint opinion by three scientific committees of the European Commission (Breitling et al. 2015) emphasizes the role of design and engineering approaches by stating that synthetic biology is “the application of science, technology and engineering to facilitate and accelerate the design, manufacture and/or modification of genetic materials in living organisms.” A report of the Secretariat of the Convention on Biological Diversity (2015) suggests that while there is no agreed international definition, the key features of synthetic biology include “the de novo synthesis of genetic material and an engineering-based approach to develop components, organisms and products.”

Proponents of synthetic biology suggest that its capabilities to design and redesign biological components and systems will address global food and energy challenges, propel industrial transformation as sustainable bio-engineered processes replace current petrochemical technologies, and offer new gene-based methods to target human medical conditions and insect-borne diseases (Church and Regis 2012; Weber and Fussenegger 2012; National Academies of Science 2013; Le Feuvre et al. 2016). The growth of synthetic biology has been boosted by a series of scientific and technological developments. These include improvements in DNA synthesis (longer fragments and higher accuracy), reduced DNA synthesis and sequencing costs, new capabilities not only to read but also to edit and rewrite the genes and cells of organisms, advances in bio-engineering design and modeling techniques, enhanced tools for biological assembly and engineering, the development of standardized biological parts, and the use of automated and data-intensive methods to speed up discovery and testing (Canton et al. 2008; Cheng and Lu 2012; Keasling 2012; Church et al. 2014; Lienert et al. 2014; Breitling and Takano 2015; Shih and Moraes 2016). The spread of synthetic biology has also been accelerated by targeted research programs and public policies, including funding by multiple federal agencies in the US (Wilson Center 2015; Si and Zhao 2016), by the UK’s network of synthetic biology research centers and its national synthetic biology roadmap (UK Synthetic Biology Roadmap Coordination Group 2012; Synthetic Biology Leadership Council 2016), by European Union projects (ERASynBio 2014), and by growing support in China (Synbiobeta 2016). Increased synthetic biology R&D investment and intellectual property acquisition by leading private sector companies in pharmaceutical, agricultural, chemical, and other sectors (OTI 2015; Carbonell et al. 2016), new business start-ups with ambitious goals such as cow-free milk or open-source insulin (Qiu 2014; Tucker 2015), community-based bio-hacking labs (Scudellari 2013), and the iGEM international synthetic biology competition (Kelwick et al. 2015) have also contributed to the emergence of the domain. At the same time, ethical, risk, equity, and other policy concerns and have been raised about the potential implications of applications of synthetic biology (Tucker and Zilinskas 2006; ETC Group 2010; OECD 2014; Engelhard 2016). These concerns have highlighted attention to the importance of responsible research and innovation in synthetic biology (Douglas and Stemerding 2013; Li et al. 2015; Shapira and Gök 2015).

In this context of rapid scientific advancement, increased public and private R&D, and stakeholder debate about the regulation and governance of synthetic biology, methods that can track the growth of research and innovation in synthetic biology are essential to inform engagement, policy deliberation, and management, and to provide evidence for decision-making. While there is a degree of high-level expert convergence on the conceptualization of synthetic biology, there are blurry boundaries between the technology in question, legacy technologies, and other new technologies that might be related to it (Nature Biotechnology 2009; Thomas 2014). There are epistemic debates about the distinctions between synthetic biology, systems biology, and genetic engineering (O’Malley et al. 2007; Calvert 2008). Synthetic biology has a legacy that extends back to the human genome project of the 1990s and early 2000s (Shapira et al. 2015) and earlier advances in understanding genes. At the same time, synthetic biology has relationships with advances in other disciplines, including engineering, biochemistry, agriculture, and informatics. Recent online discussions, hosted by the Biosafety Clearing House under the Convention on Biological Diversity (2015), demonstrate a range of perspectives from different countries and various stakeholders, about an operational definition of synthetic biology.

This paper puts forward a bibliometric approach to delineating synthetic biology. We recognize the broad notion that synthetic biology involves the design and engineering of biological components and systems at the genetic level. We also acknowledge that there is significant debate about details that affect the operationalization of a bibliometric definition of synthetic biology. We thus tread carefully through these debates, realizing that they are not yet resolved, to put forth a pragmatic strategy for creating a bibliometric definition of synthetic biology. There is relatively little work so far available on the bibliometric definition of synthetic biology, and our review of several of the definitions published to date finds them either too narrow or too expansive. We seek to contribute by refining an approach that better captures the complex scope of synthetic biology. We employ a multi-stage method, drawing from two publication indices (Web of Science and PubMed). The approach is used to identify scientific papers published in the synthetic biology domain and to trace patterns of emergence including international spread, funding, and disciplinary contributions.

The next section describes our search strategy and the steps and procedures involved. This is followed by a comparison of our results with those of other recent bibliometric definitions of synthetic biology and by an analysis of patterns of synthetic biology emergence indicated by the synthetic biology publications captured by our approach. The last part of the paper discusses the analysis and its limitations, draws conclusions and suggests lines for further work.

## Synthetic biology bibliometric search approach

Bibliometric approaches that quantitatively analyze publication, patent, and other research output indicators are commonly applied to comprehend the scale and direction of research and innovation in emerging domains of science and technology (Moed et al. 2004; Small et al. 2014). Emergent domains are intrinsically characterized by ambiguity about the sources and nature of domain novelty, contestations with established fields, uncertainty about coherence and growth trajectories, and resultant blurriness in domain delineation (Cozzens et al. 2010; Small et al. 2014; Zhang et al. 2014, 2016; Rotolo et al. 2015). Definitional problems are compounded where, as is often the case, emerging domains of science and technology span or arise from multiple disciplinary fields (Wagner, et al. 2011). Various bibliometric methods have been put forward to address the challenges of defining emergent domains, with each method having its own set of strengths and weaknesses. Indexed-based methods (using categories already defined by publication databases) promise simplicity but typically lag the emergence of new domains of science and technology (Cozzens et al. 2010). Methods based on expert-defined key words (Porter et al. 2008; Kuzhabekova and Kuzma 2014) or semi-automated searches with expert review or other tests for relevance (Mogoutov and Kahane 2007; Oldham et al. 2014) are relatively straightforward to carry out but rely on agreement among the experts and on adeptness in defining and reviewing search terms and their results. Citation or co-citation approaches, for example those that identify an agreed corpus of publications at the core of an emergent domain and then capture citations to that corpus (Zitt and Bassecoulard 2006) add in an element of peer network expertise (those researchers who reference the core and one another). However, such methods are hard to replicate (requiring access to full citation databases) and there are caveats about the interpretation of co-citations. A further method is to identify a set of journals dedicated to a domain, including through use of measures of core journal association (Leydesdorff and Zhou 2007). This method has limitations in a new domain that is interdisciplinary and where publications appear in many different journals, including disciplinary and multidisciplinary journals, alongside out-of-domain papers (Huang et al. 2010).

In addressing the bibliometric challenges of defining emerging technology domains, it has been observed that elements of these methods can effectively be combined (Glänzel 2015; Wen et al. 2017) as well as reinforced by adding contingent and iterative features (Arora et al. 2013; Huang et al. 2015). The approach put forward in this paper is thus one of pragmatic refinement, where we draw on insights from multiple methods to develop an approach to defining synthetic biology that combines a reasoned strategy, insights from existing searches, and replicability. The search approach that results should not be viewed as drawing a sharp boundary around the domain: indeed, the embryonic and interdisciplinary nature of synthetic biology means that there will be porosity in any delineation. With this in mind, the search strategy is presented as a public tool so that searches can readily be replicated, updated, modified, and refined by others, and to facilitate subsequent search term enhancements as the synthetic biology domain evolves (for an example of a similar evolution in the bibliometric search strategy for another emerging technology, see Arora et al. 2013).

Our technique for developing a bibliometric definition of synthetic biology starts with a corpus of synthetic biology benchmark papers from which we build out procedures to capture other papers that can be considered for incorporation into the synthetic biology domain. We aim to include papers that are clearly acknowledged as synthetic biology as well as papers that should be included as part of the synthetic biology domain, even though they may not explicitly use “synthetic biology” in their title, abstract or key words. We also seek to exclude papers that are in related or other fields but which are not using the concepts, methods, or sources that are associated with synthetic biology. A four-step procedure is pursued (see Table 1). First, we gather a set of benchmark synthetic biology publication records. Second, we extract additional keywords from the abstracts of these benchmark record abstract by using Natural Language Processing (NLP). Third, we test and refine these keywords, and also delineate exclusion terms. Finally, we include papers published in dedicated synthetic biology outlets. These steps are detailed below.

**Table 1:** Overview of search strategy procedure

Step	Search strategy description and sub-steps
1	Retrieve benchmark records 1.1 Download publication records searched by MeSH = “synthetic biology” from PubMed as benchmark records 1.2 Retrieve abstracts from the benchmark records
2	Extract keywords with keyword (co-)occurrence pattern and add keywords from prior studies 2.1 Extract candidate keywords from abstracts of the benchmark records 2.2 Keep high frequency keywords, drop low-frequency keywords 2.3 Combine keywords according to the keyword co-occurrence pattern 2.4 Add suggested keywords from prior studies
3	Keyword screening by noise ratio test and face validation 3.1 Measure noise ratio of each keyword 3.2 Select keywords that have low noise ratio 3.3 Extract exclusion terms by manually checking the abstract and title of the search records 3.4 Download the publication records searched by the constructed keywords set from the Web of Science. Merge with downloaded benchmark publication records (step 1.1)
4	Synthetic biology journal and special issue inclusions 4.1 Search for synthetic biology journals including special issues 4.2 Download records of the published articles in selected journal and special issues. Merge with publication records from step 3.4

### Retrieving synthetic biology benchmark records

Our search strategy begins by identifying synthetic biology benchmark publication records from PubMed, provided by the National Center for Biotechnology Information (http://www.ncbi.nlm.nih.gov/pubmed). PubMed has a set of curated publications that are expert classified as synthetic biology in the Medical Subject Heading (MeSH) terms. MeSH terms provide a controlled vocabulary that has been developed to enable more accurate searching of articles within PubMed. We used the MeSH major topic = “synthetic biology” to extract synthetic biology related papers and also to test various keywords. Our extraction of papers using the synthetic biology MeSH topic excludes records that have non-technical qualifiers such as trend, economy, or education. We used records published from 2011 to 2014 because the MeSH term for synthetic biology was not available until 2011. The benchmark set of PubMed synthetic biology publication records comprised 401 valid journal articles.

### Adding keywords from keyword (co-) occurrence analysis and prior studies

In addition to using the PubMed extracted records in our synthetic biology corpus, we generated and tested further keywords for application to other databases (such as the Web of Science). We initiated this process by extracting candidate keywords from abstracts in the PubMed synthetic biology records. We employed a natural language processing (NLP) service for keyword extraction. The service we used was AlchemyAPI, which was acquired by IBM in 2015, hence we denote as Alchemy-IBM NLP. (This service has subsequently been incorporated into the IBM Watson Developer Cloud, see: https://www.ibm.com/watson/developercloud/natural-language-understanding.html.) Alchemy-IBM NLP uses a deep learning technology for text analysis including keyword extraction. Uses of this text analysis function include studies of movie review comments and social media responses to international events (Singh et al. 2013; Simon et al. 2014). A study by Jean-Louis et al. (2014) showed that the Alchemy-IBM NLP outperforms other commercial keyword extractors in terms of precision and recall. For keyword extraction, Alchemy-IBM NLP provides a relevance score that estimates the relationship of a keyword to the context of input text. The relevance score is generated from an algorithm that combines information such as the location of words in the given text, other words around the keyword of interest, and keyword frequency. The score ranges from 0 for “unrelated” to 1 for “absolutely related.” We tested results from varying the threshold of the keyword relevance score, including manually checking the publication records associated with potential keywords. For a relevance threshold below 0.5, there were 216 potential keywords. After checking, keywords with relevance scores less than 0.5 were dropped because these were judged to bring in too many unrelated publication records. Additionally, keywords having only one occurrence were eliminated, the top 10% frequency keywords were retained, and the remaining keywords were combined and assessed according to their keyword co-occurrence pattern. This round of refinement yielded 94 keywords. Then, we added for further testing 99 keywords specified in the synthetic biology definitional paper by van Doren et al. (2013) and three keywords specified in the synthetic biology definitional paper by Oldham et al. (2012). The total pool of candidate keywords was then subject to additional processing as discussed below.

### Keyword screening using noise ratio testing and face validity review

Testing of the pool of candidate keywords was based on a comparison of the benchmark synthetic biology papers from PubMed and 1000 randomly downloaded PubMed publications published in a comparable period to the benchmark records. Using these records, two measures were calculated, *HitRatio*_*b*_ and *HitRatio*_*r*_, respectively for benchmark publications versus random publications. For each keyword *k*, we estimated the share of benchmark papers (*HitRatio*_*b,k*_) and of randomly selected papers (*HitRatio*_*r,k*_) identified by searching using this keyword. The noise ratio of keyword *k* (*NR*_*k*_) was then defined as (*HitRatio*_*r,k*_/*HitRatio*_*b,k*_). Keywords with low *NR*_*k*_ ratios are more effective than those with higher *NR*_*k*_ ratios in distinguishing benchmark records from random records. In a face validity test, we screened all keywords and manually checked identified publication records. This led us to drop 117 keywords where *NR*_*k*_ was higher than 0.1 (10% threshold). This judgment was founded on a determination that keywords above this threshold brought in too many records that were extraneous to synthetic biology. We also tested specific exclusion terms. We pursued an approach similar to that adopted by Porter et al. (2008) to formulate exclusion terms that would remove extraneous records associated with selected keywords. For example, in connection with the keyword “synthetic cell” we added exclusion terms such as “cell* phone” and “battery cell*” to avoid capturing records association with communications technology and electronics. We added terms related to “BioBricks” (sets of biological parts used to engineer biological devices and systems). We tested the term “iGEM”—the International Genetically Engineered Machine synthetic biology competition. However, we found this brought in too many extraneous records because of similar acronyms or initial characters in a range of fields. The result from these tests was a set of 21 candidate keyword terms. We excluded six of these keyword terms as they were found to add no additional publication records. These processes resulted in 15 keyword terms (14 extracted terms plus terms related to “BioBricks”) and 18 associated exclusion terms used in various combinations. Table 2 presents this keyword list, summarizes the noise test results, indicates keyword sources, and lists specific exclusion terms. Of the final set of terms, 14 present with a zero noise ratio, while “synthetic biolog*” (with the exclusion of “photosynthe*”) has a very low (0.001) noise ratio. We then transformed the keywords and exclusion terms into a consolidated search approach (Table 3). This search approach includes, but goes beyond, approaches that define synthetic biology just by that term alone or by reference to standardized parts.

**Table 2:** Synthetic biology bibliometric keyword review

Search	Keywords	Noise ratio	Origin	Specific exclusion terms
1	“synthetic *nucleotide”	0	VD	“photosynthe*”
2	“artificial nucleic acid*”	0	VD	None
3	“artificial gene* network”	0	VD	“gener*”
4	“artificial *nucleotide”	0	VD	None
5	“artificial gene* circuit*” AND “biological system”	0	New	“gener*”
6	“artificial cell”	0	VD	“cell* telephone” OR “cell* phone” OR “cell* culture” OR “logic cell*” or “fuel cell*” or “battery cell*” or “load-cell*” or “geo-synthetic cell*” or “memory cell*” or “cellular network” or “ram cell*” or “rom cell*” or “maximum cell*” OR “electrochemical cell*” OR “solar cell*”
7	“synthetic biolog*”	0.001417	Comm.	“photosynthe*”
8	“synthetic dna”	0	New	“photosynthe*”
9	“synthetic genom*”	0	VD/OD	“photosynthe*”
10	“synthetic gene*”	0	VD	“synthetic gener*” OR photosynthe*”
11	“synthetic cell”	0	VD/New	“cell* telephone” OR “cell* phone” OR “cell* culture” OR “logic cell*” or “fuel cell*” or “battery cell*” or “load-cell*” or “geo-synthetic cell*” or “memory cell*” or “cellular network” or “ram cell*” or “rom cell*” or “maximum cell*” OR “electrochemical cell*” OR “solar cell*” OR “photosynthe*”
12	“synthetic promoter”	0	New	“photosynthe*”
13	“synthetic gene* cluster”	0	VD	“photosynthe*”
14	“synthetic mammalian gene*” AND “mammalian cell”	0	New	“photosynthe*”
15	(“bio brick” or “biobrick” or “bio-brick”)	0	Expert	None

See text for discussion of noise ratio comparison of benchmark and random publications

VD = suggested by van Doren et al. (2013), OD = suggested by Oldham et al. (2012), New = newly added keywords, Expert = suggested by expert, Comm. = common keywords shared by VD, OD, and New

**Table 3:** Consolidated keyword terms for synthetic biology search strategy (in Web of Science)

Search strategy—synthetic biology inclusion and exclusion terms	
(((TS = (“synthetic biolog*” OR “synthetic dna” OR “synthetic genom*” OR “synthetic *nucleotide” OR “synthetic promoter” OR “synthetic gene* cluster”) NOT TS = (“photosynthe*”)) OR (TS = (“synthetic mammalian gene*” AND “mammalian cell”) NOT TS = “photosynthe*”) OR (TS = “synthetic gene*” NOT TS = (“synthetic gener*” OR “photosynthe*”)) OR (TS = (“artificial gene* network” OR (“artificial gene* circuit*” AND “biological system”)) NOT TS = “gener*”) OR (TS = (“artificial cell”) NOT TS = (“cell* telephone” OR “cell* phone” OR “cell* culture” OR “logic cell*” or “fuel cell*” or “battery cell*” or “load-cell*” or “geo-synthetic cell*” or “memory cell*” or “cellular network” or “ram cell*” or “rom cell*” or “maximum cell*” OR “electrochemical cell*” OR “solar cell*”)) OR (TS = (“synthetic cell”) NOT TS = (“cell* telephone” OR “cell* phone” OR “cell* culture” OR “logic cell*” or “fuel cell*” or “battery cell*” or “load-cell*” or “geo-synthetic cell*” or “memory cell*” or “cellular network” or “ram cell*” or “rom cell*” or “maximum cell*” OR “electrochemical cell*” OR “solar cell*” OR “photosynthe*”)) OR (TS = (“artificial nucleic acid*” OR “artificial *nucleotide”)) OR (TS = (“bio brick” or “biobrick” or “bio-brick”))))	

This definition is applied directly into the advanced search feature of the Web of Science. It does not incorporate the additional synthetic biology journal search strategies described in the paper (see also Table 4)

### Synthetic biology journal and special issue inclusions

In a further refinement step, we also included papers published in curated synthetic biology journals and special synthetic biology issues of general journals (see Table 3). We added this step to ensure the inclusion of papers that editors have determined are within the synthetic biology domain. This set included *PLoS ONE* curated synthetic biology articles from http://collections.plos.org/s/synbio; this source yielded 185 publication records. We added all publication records from the synthetic biology specialty journal *ACS Synthetic Biology*. We also added publication records in special issues of general journals with a focus on synthetic biology, including *Trends in Biotechnology* 33(2), *ACM Journal of Emerging Technologies in Computing Systems* 11(3), *Biochimica et BioPhusica Acta*—*Gene Regulatory Mechanisms* 1839(10), *Biochimica et Biophysica Acta*—*Bioenergetics* 1837(9), *Natural Computing* 12(4), *Chemical Engineering Science* 103, *FEBS Letters* 586(15), and *Acta Biotheoretica* 58(4). These journals and special issues yielded 558 records (Table 4).

**Table 4:** Synthetic biology bibliometric search: journal inclusions

Journal or journal special issue
PLOSONE curated synthetic biology articles from http://collections.plos.org/s/synbio
ACS Synthetic Biology
Trends in Biotechnology volume 33(2)
ACM Journal on Emerging Technologies in Computing Systems volume 11(3)
Biochimica et Biophysica Acta-Gene Regulatory Mechanisms volume 1839(10)
Biochimica et Biophysica Acta-Bioenergetics volume 1837(9)
Natural Computing volume 12(4)
Chemical Engineering Science volume 103
FEBS Letters volume 586(15)
Acta Biotheoretica volume 58(4)

Where applicable, journal issue number is in parenthesis

### Web of Science search and data cleaning

We applied the consolidated search approach (including exclusion terms) and the journal search strategy to publications recorded in the Web of Science (WoS) for the period 2000–2015 in Science Citation Index Expanded (SCI-Expanded), Social Sciences Citation Index (SSCI), Conference Proceedings Citation Index-Science (CPCI-S), and Conference Proceedings Citation Index-Social Science and Humanities (CCPI-SSH). The gross worldwide number of records obtained by applying this search strategy (in May 2016) was 8412. VantagePoint text mining software was then used for record cleaning. This software is customized to enable the data cleaning, text mining, and analysis of bibliometric publication records (https://www.thevantagepoint.com/). After removing duplicate records, including early conference papers subsequently published with the same title, abstract and authors as articles, our synthetic biology publication dataset comprised 8064 publication records.

### Comparison with other bibliometric definitions of synthetic biology

As indicated in the introduction, there is debate about the operational definition of synthetic biology, i.e. what should be included (and excluded). Given our focus on the bibliometric definition of synthetic biology, we assessed our search strategy against three other comparable studies. The first of these studies, by Oldham et al. (2012), in acknowledging the complexity of defining synthetic biology, explicitly eschewed a multifaceted approach, choosing instead to use a simple and restricted search strategy. This restricted strategy used only four terms to bibliometrically define synthetic biology: “synthetic biology”, “synthetic genomics”, “synthetic genome”, and “synthetic genomes”. A subsequent study, by Raimbault et al. (2016) identified a set of core articles using a topic search for synthetic biology, examined relevant keywords, and using expert review added 11 additional search terms. This approach added refinements to the Oldham et al. strategy, for example by adding terms such as “synthetic gene network” or “synthetic gene circuits.” Raimbault et al. also added several other terms, including “standard biological parts” and “minimal cells”.

In a third study, Hu and Rousseau (2015), from WoS articles using the term “synthetic biology”, extracted two types of keywords, analyzed the frequencies of those key words, correlated the most common terms with MeSH subject definitions and Wikipedia descriptions, and verified their list of most-used terms with a field expert. The outcome added a further 23 search terms to the core term of “synthetic biology”. There is commonality between all three definitions in the use of term variations related to “synthetic gene*” and “synthetic genomics.” Hu and Rousseau’s insertion of “biobrick*” also has a correspondence (see Shetty et al. 2008) with the inclusion of “standard biological parts” by Raimbault and colleagues. However, there are differences. For example, Hu and Rousseau explicitly include “DNA nanotechnology”—a topic that tends to focus more on DNA’s physical and chemical features than on its genetic aspects (Nature 2016). Hu and Rousseau also include the terms “protein engineering” and “metabolic engineering”. Research in these two established fields has typically been oriented towards enhancing existing biological mechanisms, for instance to develop proteins and enzymes, while synthetic biology arguably has an orientation to adding new biological components to make mechanisms work differently. While there are emerging overlaps between these fields and synthetic biology (Jungmann et al. 2008; Keasling 2012; Li 2012; Yadav et al. 2012), the specific inclusion of these terms (and others, such as “protein design”) means that Hu and Rousseau’s bibliometric definition of synthetic biology extends rather broadly.

The three comparison synthetic biology search strategies discussed here, plus our own, each use the WoS as the source of publication records. However, different publication periods and end dates are used and there are also variations in the WoS databases searched and types of publications extracted. Hence, to compare these studies, we applied the respective bibliometric search strategies to identify articles published in WoS SCI-Expanded and SSCI between the years 2000 and 2015. The search results were organized and cleaned in VantagePoint, using common methods (for example, to eliminate duplicates and to ascertain keywords). This standardized comparison yielded significant differences in the number of records captured by each search (see Table 5). The “minimalist” approach of Oldham and colleagues to defining synthetic biology returned nearly 2400 articles. The synthetic biology search strategy of Raimbault and colleagues garnered only 380 more articles, notwithstanding the additional keywords added. In contrast, Hu and Rousseau’s broad definition returned a much larger set comprising over 18,100 articles. The synthetic biology search strategy that we put forward in this paper (see prior section) yielded about 5400 articles.

**Table 5:** Comparison of four bibliometric definitions of synthetic biology

Study	Search approach	Study findings		Standardized search, 2000–2015
		Period of study and data source	Records identified	WoS articles
Oldham et al. (2012)	Topic search for synthetic biology and synthetic genomics Search strategy: TS = (“synthetic biology” OR “synthetic genomics” OR “synthetic genome” OR “synthetic genomes”)	1990–2011; WoS (including articles, proceedings, and other publications)	1255 publications 5995 forward citations	2384
Raimbault et al. (2016)	Core articles identified with topic search for synthetic biology (*N* = 1198 in March 2012) and 30 most specific keywords extracted. Expert review leads to final set of key search terms, adding about 500 articles. Search strategy: TS = (“synthetic biology” OR “synthetic gene network” OR “standard biological parts” OR “artificial genetic system” OR “synthetic genom*” OR “synthetic gene circuits” OR “minimal cells” OR “synthetic circuits” OR “synthetic networks” OR “synthetic cells” OR “minimal genome” OR “artificial gene networks”)	All years to 2012 (May); WoS	1698 articles	2764
Hu and Rousseau (2015)	Core articles identified with topic search for synthetic biology (*N* = 1333 in January 2014) and keywords extracted. List of most used terms selected using MeSH with validation by a field expert. Search term expanded. Search strategy: TS = (“synthetic biology” OR “synthetic gene network*” OR biobrick* OR “protein design*” OR “genetic circuit*” OR “gene regulatory network*” OR “cell-free protein synthes*” OR “metabolic engineering” OR “protein engineering” OR “promoter engineering” OR “DNA assembly” OR “RNA engineering biosensors” OR “multipart DNA assembly” OR “sequential circuits” OR “benchmark synthetic circuits” OR “DNA nanotechnology” OR “human artificial chromosome” OR “synthetic promoters” OR “transcriptional circuits” OR “abstract genetic regulatory network*” OR “gene assembly” OR “post-transcriptional regulation” OR “engineered proteins” OR “cell-free gene circuits”)	2000–2013; WoS SCI-EXPANDED, SSCI, A&HCI, CPCI-S, CPCI-SSH; Document type: articles	13,836 articles	18,174
Shapira et al. (current bibliometric search strategy)	From core benchmark publications in synthetic biology, keywords extracted and refined, adding papers published in dedicated synthetic biology journals (see text and Tables 3 and 4, this paper)	2000–2015; WoS SCI-EXPANDED, SSCI, CPCI-S, CPCI-SSH; Document type: all	8064 publications	5806

Standardized search: WoS SCI and SSCI, 2000–2015, articles, accessed in May 2016 and cleaned in VantagePoint

*WoS* Web of Science, *SCI* Science Citation Index, *SSCI* Social Sciences Citation Index, *A&HCI* Arts and Humanities Citation Index, *CPCI-S* Conference Proceedings Citation Index-Science, *CPCI-SSH* Conference Proceedings Citation Index-Social Science and Humanities, *TS* topic search

For a systematic comparison of the four approaches, we profiled the top author keywords for the articles returned by each definition. Overall, our definition has a profile for the highest ranked author keywords comparable to that found in the Oldham and Raimbault definitions. “Synthetic biology”—the most frequent keyword captured by all three definitions—was about 6.5 times more likely to appear than the next term, which was metabolic engineering. “System biology” was third ranked in the Oldham and Raimbault definitions, with “Synthetic gene” and “Escherichia coli” ranked above “System biology” in our definition. The search strategy that we put forward yields more articles than the two other definitions, yet still returns records that capture features of synthetic biology. The Hu and Rousseau definition yields a noticeably different ordering of top keywords. In their definition, “Engineered proteins” is the most frequently keyword, appearing about 1.9 times more frequently than third-ranked “Synthetic biology.” “Metabolic engineering” ranks second in Hu and Rousseau, appearing 1.5 times more frequently than “Synthetic biology.” Other keywords containing the term “Protein” appear more than in the other three definitions. Also, the Hu and Rousseau definition yields a rather longer tail distribution of the keywords than the three other definitions (including our definition).

As with other emerging technologies where boundaries are blurry, there is not necessarily a single way to bibliometrically define synthetic biology. Each method has its own mix of strengths and weaknesses. The Oldham “minimalist” definition, with the use of just four search terms, appears to have relatively high precision in terms of the limited set of papers retrieved. However, Oldham also performs weakly in terms of recall in that it fails to capture synthetic biology papers whose authors do not explicitly use the term “synthetic biology” or refer to “synthetic genome” or one of its variations. While Oldham and colleagues succeed in avoiding false positives, arguably they significantly undercount the breadth and scope of the synthetic biology domain. Raimbault et al. seek to address the recall problem by introducing additional keywords, but those added terms lead to only a small recall increment in part because most of the new terms are modest variants on Oldham’s. Conversely, the Hu and Rousseau strategy captures a much wider range of topics. Recall is significantly increased, but at the expense of precision as this definition raises concerns that papers are being captured that are more related to general or predecessor biological concepts rather than to topics that are more directly connected with the contemporary thrust of synthetic biology. The analysis and comparison indicates that our definition is focused on the synthetic biology domain while capturing more features of synthetic biology than “minimalist” definitions. This is not a “golden mean” compromise, i.e. we do not arbitrarily split the difference between two boundary positions. Rather, we derive our definition through a process that builds on the terms and associations evident among those who publish and curate in the synthetic biology domain as well as reviewing terms suggested by those who have previously formulated bibliometric synthetic biology field definitions. The process considers candidate keywords identified in recognized synthetic biology publications, through co-occurrence analysis, and in prior studies; screens and tests keywords before acceptance, incorporating exclusion terms; and then merges externally curated publication records. While this search process identified a significant number of potential new keywords, the subsequent screening and exclusion process ensured that only those new terms that measured up well in terms of precision were retained. Recall and precision also received reinforcement by the hybrid addition of curated synthetic biology journals and collections. Overall, the method put forward in the paper has reasonable recall and precision, avoiding the potential under- and over-counts of other available approaches: this is important in appropriately assessing the scale and scope of the emergent synthetic biology domain—the topic that we now turn to in the next part of the paper.

## Analysis of results

The purpose of our synthetic biology bibliometric definition and search approach is to provide a tool that can be used to track developments and patterns in this research domain. In this section, we present the results of analyses using the publications dataset derived from the search. We look at the growth of synthetic biology publications over time and by leading countries and explore international author collaborations. We then explore the scientific disciplines that underpin the emergence of synthetic biology, including analysis of the profile of synthetic biology on the map of science. Finally, we examine the emergence of research sponsorship in the development of synthetic biology and investigate insights provided by funding acknowledgements information.

### Synthetic biology publications and collaborations by author countries

**Fig. 1:**
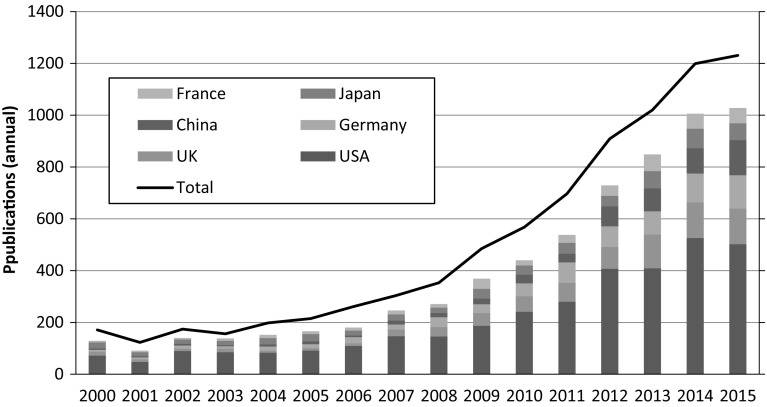
Synthetic biology publications, worldwide and leading countries by author affiliations. *Source*: Synthetic biology publications, 2000–2015 (*N* = 8064 WoS records). See text for search strategy. *Line graph* depicts worldwide annual publications. *Bar chart* depicts annual publications for the six leading countries by total publication output

The synthetic biology search strategy that we presented in the prior section finds that annual worldwide synthetic biology publication output grew from an average of about 170 publications per year from 2000 to 2005 to well over 1200 publications in 2015. Here, we use the full cleaned dataset of WoS publication records, including articles, reviews, editorials, proceedings, conference papers, and book chapters. Synthetic biology publication output noticeably accelerated from 2008 (Fig. 1). Our synthetic biology publication dataset includes publications by more than 25,000 authors at 3700 organizations located in 79 countries. Yet, while researchers from many countries are now involved in publishing work on synthetic biology, it is evident that the bulk of research output derives from a sub-set of countries. The US is the leading country by number of synthetic biology publications, with US-based authors contributing to 42.4% of identified worldwide 2000–2015 publications. Authors based in the UK contributed to 10.1% of synthetic biology publications over the same period, followed by Germany (8.9%), China (7.0%) and Japan (6.8%). (Percentage totals calculated by authors add up to more than 100% if all countries are included, as some publications have authors from two or more countries.) Publications with US, UK, German, and Japanese authors more than tripled from 2008 to 2015 while publications with at least one author based in China grew by almost a factor of eight over this period (Fig. 1). The rapid rise of China, joining US, European and Japanese researchers engaged in synthetic biology research, is notable. This reflects the massive expansion of research investment, scientific human capital, and publication output in China in recent years (Bound et al. 2013; Zhou 2015) and its policy focus to develop leading-edge capabilities in emerging fields, including in synthetic biology (Pei et al. 2011; National Academy of Sciences 2013, 17–19; Li and Shapira 2015).

**Fig. 2:**
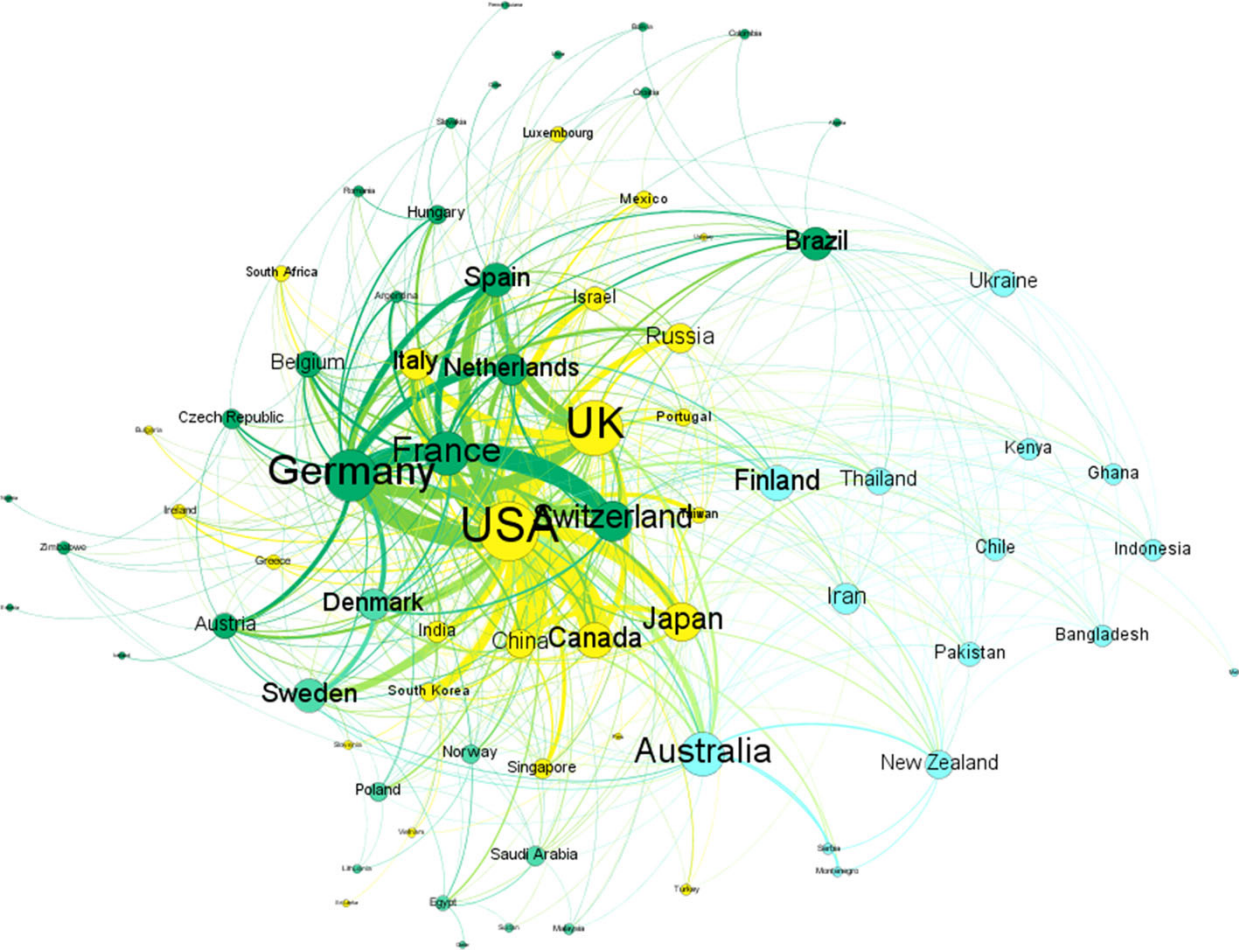
Co-author collaboration network by country, synthetic biology publications, 2000–2015. *Note*: Visualization with Gephi (http://gephi.github.io/) using ForceAlas2, clustered by modularity. Countries without international collaborations are not included. *Source*: Synthetic biology publications, 2000–2015.

While we observe the growth of national synthetic biology research publication outputs in key countries, it is also apparent that the performance of science intrinsically crosses national boundaries (Leydesdorff et al. 2013). This is evident in the international co-authorship patterns associated with synthetic biology research. However, there are variations in international co-authorship patterns. For the US, 29% of synthetic biology publications with US authors also have authors from other countries, while for China and Japan the comparable percentages are 36 and 27% respectively. International co-authorship levels are higher for European authors, with 55% of UK synthetic biology publications co-authored with researchers from other countries, while for Germany the corresponding percentage is 61%. For US authors of synthetic biology publications, international co-authors are most commonly located in the UK, China and Canada (with authors from each of these countries co-authoring more than 100 papers with US counterparts). UK international synthetic biology co-authorships are most frequently with US colleagues, then with researchers from Germany, Italy, Canada, and France. German international synthetic biology co-authorships are also most commonly with the US, followed by Switzerland, the UK, the Netherlands and France. Chinese international synthetic biology co-authorships are dominated by collaborations with the US, with Japan and the UK representing somewhat fewer international co-authorships. We depict these and other collaboration patterns among countries through an international co-author network visualization (Fig. 2) where node size is proportional to country output and linkage thickness is proportional to the number of co-authorships between country pairs. The major countries that produce synthetic biology publications can be clustered into three groups: US–UK–Japan–Canada–China (colored in yellow); Germany–France–Switzerland (green); and Australia and Finland (blue). Articles authored by researchers in these three clusters comprise 81% of our global synthetic biology publication dataset.

### Scientific disciplines of synthetic biology

Synthetic biology is frequently described as an interdisciplinary research domain with contributions from biology, engineering, chemistry, computer science and other disciplines (National Academy of Sciences 2010; Cheng and Lu 2012). Yet, as broached in the opening part of the paper, there are also debates about the fields and specialties that underpin synthetic biology. Such discussions are important not only for definitional purposes but also because they suggest different trajectories for the emergence of synthetic biology (Vincent 2013).

To offer further insights on the nature of the disciplines that are contributing to synthetic biology, we draw on our synthetic biology publications dataset to analyze the subject categories associated with these records. Each publication is assigned to at least one of the more than 250 subject categories designated by the Web of Science based on citation patterns and judgement. The assignment of subject categories is based on the source journal or book where the publication is placed. One or more subject categories can be allocated: of the more than 1800 publication sources in our synthetic biology dataset, 44% are assigned one subject category, 36% have two subject categories, 18% have three or four subject categories, and a handful (about 1%) have five or more subject categories. The assignment of subject categories to a publication record does not necessarily represent the research disciplines of all the individual authors. However, when aggregated for many publications, the distribution of subject categories, and changes in the distribution of time, can signal broad research field trends. For example, analysis of our synthetic biology publications dataset indicates that the publications in the domain were distributed over about 60 subject categories in the first part of the 2000s. While there is change in WoS subject categories from time to time, and new journals enter with different subject category distributions, it is noticeable that by 2015, publications in the synthetic biology domain were distributed across 109 subject categories, suggesting disciplinary dispersion.

Yet, while there is a broad spread, it is also apparent that the bulk of synthetic biology publications are assigned to sources within a rather smaller number of subject categories. An analysis of the top ten WoS subject categories in the synthetic biology publication dataset shows the dominance of three categories: “biochemistry and molecular biology”, “biotechnology and applied microbiology”, and “biochemical research methods” (Table 6). Since 2010, publications assigned to “biochemical research methods” have more than doubled in their share, with a major contributor to growth in this category being the journal *ACS Synthetic Biology* (430 records) which first started publishing in 2012. In the category multi-disciplinary science, a boost is given by the entry of *PlosOne*, where the first publications in our dataset are recorded in 2006. We note that while the established biology fields of “cell biology,” “microbiology,” and “genetics and heredity” are in the top ten subject categories, they take a smaller position in the composition of synthetic biology research fields. “Mathematical and computational biology” increased its role from the first part to the second part of the 2000s, but then saw a more recent downward trend in share, possibly because synthetic biology papers with mathematical modeling and computer simulation can now also find outlets in multidisciplinary and other research methods categories.

**Table 6:** Top 10 Web of Science subject categories, synthetic biology publications. *Source*: Synthetic biology publications, 2000–2015 (*N* = 8064)

Publication years	2000–2005	2006–2010	2011–2015
Publications	1037	1971	5056
*Subject categories*	*Percent of total publications*		
Biochemistry and molecular biology	38.1	25.8	19.0
Biotechnology and applied microbiology	16.2	19.0	21.1
Biochemical research methods	8.0	9.2	19.8
Multidisciplinary sciences	5.9	9.8	11.5
Chemistry, multidisciplinary	7.5	7.5	6.9
Cell biology	7.5	5.4	4.1
Biophysics	7.0	4.0	4.5
Microbiology	4.1	4.1	4.7
Genetics and heredity	6.3	4.6	3.4
Mathematical and computational biology	1.3	5.6	3.7

Total number of Web of Science subject categories in synthetic biology publications = 178. Percentages for all subject categories sum to more than 100% as publications may be associated with more than one journal or book subject category

**Fig. 3:**
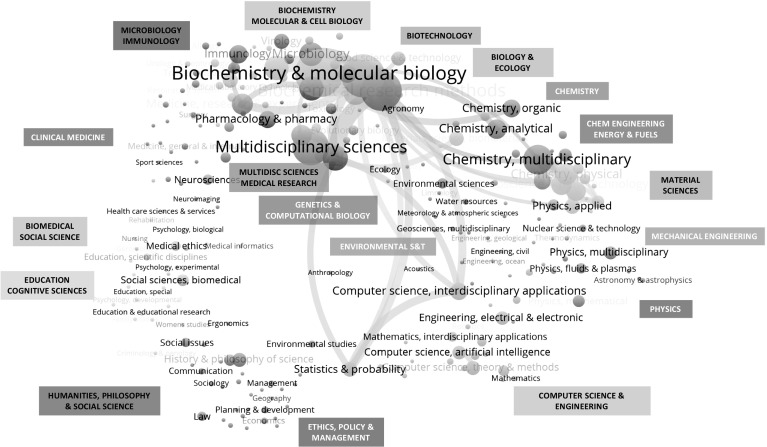
Profile of synthetic biology research by clusters and subjects, arrayed on the map of science. *Source*: Synthetic biology articles (from WoS SCI-EXPANDED and SSCI), 2000–2015 (*N* = 5806). Map of science method from Carley et al. (2016), using VOSviewer (Van Eck and Waltman 2016), with customization of 2015 WoS 18-category macro-discipline labels for synthetic biology

In an extension of the analysis, we layer the synthetic biology publication dataset onto a base map of science (Porter and Rafols 2009; Rafols et al. 2010). The base map is a representation of the underlying structure of science, based on the analysis of co-citation patterns among subject categories of all WoS-indexed journals. We draw on the enhanced overlay science base map and the clustering of subject categories into 18 macro-disciplines constructed with 2015 WoS journal data by Carley et al. (2016) and visualized with VOSviewer (van Eck and Waltman 2016). The VOSviewer clustering method and algorithm is used (van Eck and Waltman 2010). The map visualizes the distance and intensity of co-citations between corresponding subject categories. Each node represents individual WoS subject categories, with colors used to depict subject categories within the same macro-discipline group. The subject categories that are assigned to synthetic biology articles in our publication dataset are matched to the 18 macro-disciplines and displayed on the base map (Fig. 3). We use articles (published 2000–2015, *N* = 5806) in the synthetic biology publication dataset as this matches the journal article and subject category analysis method used by Carley and colleagues. In our array of synthetic biology research on the map of science, the node size is proportional to the number of articles in that subject category. While synthetic biology spreads broadly across the macro-disciplines and subject categories in natural sciences and engineering, with representation in the humanities and social science, we observe concentrations in three clusters. The largest cluster (by total papers for the 2000 to 2015 period) is “biochemistry, and molecular and cell biology.” This is followed by “chemistry” (which includes the “biochemical research methods” category) and “biotechnology” (which includes “plant sciences”). A parallel analysis of publications in the leading macro-disciplines of synthetic biology over time (see Table 7) indicates a redistribution of relative emphasis among the top three clusters. In the context of absolute increases in paper outputs for all three clusters from 2000 through to 2015, the “chemistry” macro-discipline (driven particularly by growth in “biochemical research methods”) took a larger relative share of publications over the recent 5 year period (2011–2015). Relative growth in this most recent period was also seen in publications in the “biotechnology” cluster.

**Table 7:** Top 10 macro-discipline of synthetic biology *Source*: Synthetic biology publications, 2000–2015 (*N* = 8064)

Publication years		2000–2005	2006–2010	2011–2015
Publications		1037	1971	5056
*Macro*-*disciplines*	*SCs*	*Percent of total publications*		
Biochemistry; Molecular and Cell Biology	14	47.0	31.4	22.9
Chemistry	10	20.4	19.8	29.1
Biotechnology	12	20.6	22.5	24.4
Multidisciplinary Sciences; Medical Research	6	13.9	12.8	14.3
Genetics; Computational Biology	9	13.9	14.4	10.1
Material Sciences	17	5.7	8.6	7.6
Microbiology; Immunology	8	10.2	6.5	6.6
Computer Science and Engineering	30	3.6	8.6	7.0
Biology and Ecology	10	3.3	4.0	2.7
Chemical Engineering; Energy and Fuels	7	0.5	1.2	3.0

SCs = Number of Web of Science subject categories in macro-discipline. Method of assignment of subject categories to macro-disciplines based on Carley et al. (2016). Percentages for all macro-disciplines sum to more than 100% as publications may be associated with more than one journal or book subject category

The profile of synthetic biology research on the map of science also depicts groupings of synthetic biology papers that are found among the clusters of “multidisciplinary sciences” (including “research and experimental medicine”), “genetics and computational biology,” “materials science” (which includes “nanoscience and nanotechnology” and “biomedical engineering”), and “microbiology and immunology.” Subjects in the “computer science and engineering” cluster (including “computer science, interdisciplinary applications” and “statistics and probability”) are noticeably linked to subjects in the core synthetic biology clusters, including “biochemistry and molecular biology,” “biochemical research methods,” and “genetics and heredity.” Just over 280 papers (or 4.9% of our dataset of synthetic biology papers) are in subject categories with explicit reference to “engineering”, led by the three largest categories of biomedical, chemical, and electrical and electronic engineering. If papers that use engineering (or a variations such as metabolic or genome engineering) are added to the papers in engineering subject categories (with duplicates removed), we find about 625 papers (10.8% of the dataset). Papers in engineering subject classifications or which use engineering as a key word are arrayed across multiple macro-disciplines on the profile map of synthetic biology. If papers that use terms such as design or assembly were also identified, the number of synthetic biology papers explicitly referring to engineering concepts would be higher and new variations in the distribution might be observed—although another study would be required to fully elucidate, test, and analyze this proposition. About 165, or 2.9 percent, of the articles in our dataset that raise synthetic biology topics are papers with subject categories in the humanities and social sciences. The overlap map shows visible nodes in the “history and philosophy of science,” “social science, biomedical,” “ethics,” “medical ethics,” “social issues,” and “law.”

### Research sponsorship

Multiple factors, structures, and dynamics underpin the practices and trajectories of science and, in particular, the emergence of new scientific domains (Merz and Sormani 2015). One of the key enabling elements, especially in the contemporary practice of both public and private science and its growth, is the availability of funding to support researcher time, laboratories, and the various other costs and overheads associated with scientific research (Shrum et al. 2007; Kennedy 2012). Individual researchers and teams can (and do) pioneer new scientific approaches and methods, by using already available resources and, where necessary, seeking added funding in researcher-initiated open requests for proposals. However, an uptick in support from the sponsors of research is generally indispensable for an emerging field of scientific inquiry to speedily “take-off”, to initiate new research projects, acquire recognition, attract and train new researchers, and build a critical mass of interdisciplinary and institutional collaborators. Increased support for an emerging field can draw on existing funding sources and programs or, as is often the case, through the introduction of dedicated funding calls and programs. There is debate about the relationships between additional tranches of research funding for science and the utility of the knowledge that results (Sarewitz 2016). Nonetheless, it is apparent that whether and how an emerging domain attracts support, including what kind of research is performed and its scale and scope, is intrinsically intertwined with the priorities and policies of the bodies that support research and the organizational and governance landscapes within which these research sponsors operate (Stefan 2012). This includes extramural scientific research funded by research councils, government agencies, foundations, and corporations, as well as internal or core research support from within research-performing organizations.

In tracing the precursors of synthetic biology, pointers can be directed to the early 1960s and early work on molecular network cell regulation, molecular cloning in the 1970s and 1980s, and the growth of genomics and systems biology in the 1990s (Campos 2009; Cameron et al. 2014). From 2000 to 2003, denoted by Cameron and colleagues as synthetic biology’s “foundational years”, engineering concepts of genetic circuits, networks, and switches advanced significantly. The key funders of this early millennium synthetic biology research included the US Defense Advanced Research Projects Agency (DARPA), the US Office of Naval Research (ONR), the US National Science Foundation (NSF), and the US National Institutes of Health (NIH). Early research funding was also provided by US and European foundations, and Canadian, European and other US research funders. Interest in synthetic biology, both from researchers and from funding bodies, took off from around 2004. That year saw the first major international synthetic biology conference, held in the US (MIT 2004; Campos 2009), and the founding of the synthetic biology student competition that is now the International Genetically Engineered Machine (iGEM) competition (Smolke 2009). The concept of open-access standardized biological parts was advanced in this period, with the establishment of the BioBricks Foundation in 2006 (Minssen and Wested 2015). From 2004 to 2006 NSF ramped up funding for synthetic biology research, leading to the formation of the multi-partner Synthetic Biology Engineering Research Center (SynBERC) which was awarded more than $37 million in 2006 under an NSF cooperative agreement with the University of California, Berkeley (NSF 2006; Si and Zhao 2016). In 2005, the Alfred P. Sloan Foundation sponsored an initiative to examine the risk, societal, ethical and governance implications of synthetic biology, awarding nearly $10 million through to 2014 (Sloan Foundation 2016). In 2008, NSF awarded nearly $34 m to a second Engineering Research Center, this to the Center for Biorenewable Chemicals (CBiRC) at Iowa State University (NSF 2008). Overall, from 2008 through to 2014, the Wilson Center (2015) identifies about $820 million of US public research funding as allocated to synthetic biology; of this, three-fifths was sponsored by DARPA, with funding from NSF, NIH, the Department of Energy (DOE), and other Department of Defense (DOD) agencies comprising much of the balance. The Wilson Center estimates less than two percent of this funding has gone to research on risk, ethical, legal, and societal issues.

In Europe, research support explicitly for the development of synthetic biology began to expand in the mid-2000s. In the UK, the Biotechnology and Biological Sciences Research Council (BBSRC) sponsored multi-university Networks in Synthetic Biology beginning in 2007, while the Engineering and Physical Sciences Research Council (EPSRC) awarded £4.7 m in 2009 to a Centre for Synthetic Biology and Innovation (CSynBi) at Imperial College London. UK research sponsorship of synthetic biology grew to upwards of £200 million by 2015, with a further six university BBSRC “Synthetic Biology Research Centres (SBRCs)” established in 2013 and 2014, along with support for synthetic biology doctoral training, academic-industry networks, commercialization, and related research and translational projects (Shapira and Gök 2015; Clarke and Kitney 2016). In the mid-to-late 2000s, a 10-year €60 million university research investment in synthetic biology was reported in the Netherlands, the Swiss National Science Foundation (SNSF) expanded synthetic biology research funding, and smaller synthetic biology research programs (in addition to general research funding) were initiated in Germany, France, and some other European countries (Wilson Center 2010; Meyer 2013; Pei et al. 2012). The European Union enlarged its funding of synthetic biology research from 2005 onwards, awarding an estimated €86 million to synthetic biology under its 7th Framework Programme (2007–2013), with support continuing under the Horizon 2020 (2014–2020) Research and Innovation Programme (ERASynbio 2014). There are no updated measures of European research funding for risk, ethical, legal and societal implications of synthetic biology (beyond a 2010 estimate of about 2% by the Wilson Center), although some support for responsible research and innovation has been embedded into the UK SBRCs as well as in projects sponsored by the European Union (Stemerding and Rerimassie 2013; Hagen 2016). Among other countries (see also OECD 2014), synthetic biology research funding in China began to pick up towards the end of the 2000s, with funding from the National Natural Science Foundation of China (NSFC) and other governmental R&D and technology programs (Yang 2009; Pei et al. 2011; Chen and Feng 2016).

With the global expansion of research activities and outputs in synthetic biology in recent years, a systematic approach to tracking the linkages between research funders and research outputs is needed. This can now be done at a large scale by data mining funding acknowledgments information that, since mid-2008, is available in the records of journal publications in the WoS. The analysis of funding acknowledgements information offers broad insights about who is sponsoring research, what research gets funded and how it is carried out (Shapira and Wang 2010). There are caveats. For example, not all awards are acknowledged (although sponsors are increasingly making this a requirement), the recording of social science and humanities awards is lower than for natural sciences and engineering, there are variations in the reporting of the names and programs of funding agencies, and work supported through ongoing institutional resources may not be acknowledged as there is no dedicated funding award (Wang and Shapira 2011; Tang et al. 2016). That said, of the 4250 journal articles in our synthetic biology database published between 2009 and 2015, a relatively high share—3405 or 80.1% of these papers—acknowledge one or more funders. The percentage of articles with funding acknowledgements in our synthetic biology publications dataset rose from 68% in 2009 (soon after the WoS started to report acknowledgements) to 85% in 2015. In short, in the 7 year time period that we analyze, four in five synthetic biology papers report funding acknowledgements, and the proportion is higher for more recent papers.

For the synthetic biology papers with funding information, more than 5000 variations in sponsor names are listed. The types of sponsor acknowledged included research councils, national and regional government agencies and programs, universities and research centers, foundations, corporations, international agencies and international research partnerships, and career advancement, fellowship, and mobility awards. We undertook an iterative cleaning process, using VantagePoint to develop a master thesaurus of variants of sponsor names, abbreviations, and acronyms and coupled this with manual review and verification. Judgement was exercised in how to combine (or separate) certain sponsors. For example, we separated out the European Research Council which has a distinct operational mode in selecting research for funding from the European Union with its multiple framework, collaborative research, and developmental programs. We also distinguished individual key mission funding agencies within the US Department of Defense, and disaggregated prominent Chinese government funding programs. After three rounds of cleaning, re-aggregation, and review, just under 2800 cleaned sponsor names emerged. On average, each funded paper acknowledged 2.6 sponsors, with 60% of the papers acknowledging between two to four sponsors. Multiple research sponsorship can reflect acknowledgement to a research line, team or center that has attracted more than one research sponsor and, since synthetic biology papers are typically multi-authored, acknowledgement to the mix of author funding sources.

**Fig. 4:**
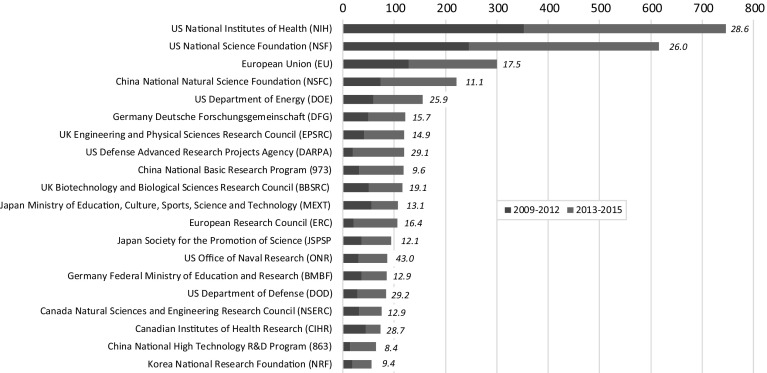
Top 20 research sponsors of synthetic biology articles. *Source*: Synthetic biology articles, 2009–2015, with one or more funding acknowledgements (*N* = 3392). *Y*-axis depicts number of papers acknowledging research sponsor. A paper may have more than one funding acknowledgement. *Italicized number to right of each bar* reports average WoS citations per paper to papers acknowledging that research sponsor

Although there is a wide global spread of research sponsors of synthetic biology, there is also concentration among a subset of sponsors. While the available data does not provide funding amounts, it does allow analysis of the distribution of acknowledgements to sponsors. A cluster of 30 research sponsors is acknowledged in 74.7% of the funded articles, within which a top group of 20 research sponsors is acknowledged in 70.6% of the funded synthetic biology articles. By the number of WoS synthetic biology articles published between 2009 and 2005 with funding acknowledgment information, two leading sponsors stand out (Fig. 4). NIH is acknowledged in about 750 articles, with NSF acknowledged in just over 615 articles. These two US sponsors are followed by the European Union, NSFC (China), and the US DOE. Overall, the US has six funders among the top 20 global sponsors of synthetic biology research, China has three, Canada, Germany, Japan, the UK, and the European Union each have two, while South Korea has one. By supranational regional bloc, eight funders among the top 20 global sponsors of synthetic biology research are located in North America, and six each in Europe and Asia, although the top US research sponsors have supported more synthetic biology articles to date than the combined total of the 14 top-twenty sponsors in other countries. Represented among the leading US synthetic biology research sponsors are DARPA, ONR, and DOD. For China, in addition to NSFC, the top sponsor group includes two programs of the Ministry of Science and Technology: the 973 National Basic Research Program (multidisciplinary basic research) and the 863 National High-Tech R&D Program (research on potentially commercializable technologies). Two interrelated European supra-national bodies are in the top group: the European Union’s research and innovation programs and the European Research Council. With the exception of Japan’s Ministry of Education, Culture and Sport (MEXT) and the Canadian Institutes of Health Research (CIHR), all other sponsors in the top twenty group funded more papers in the most recent 3-year period (2013–2015) than in the prior 4 year period (2009–2012). Comparing the most recent with the prior period, the greatest relative growth in funded articles was supported by DARPA, the European Research Council, and China’s 863 R&D Program.

Looking deeper into the ranks of the sponsors of synthetic biology research (by numbers of papers with funding acknowledgements), the great majority of funders in the top 50 are public research councils or government agencies. Just outside the top twenty group is the Human Frontier Science Program, an international partnership of 14 countries (including the US and six European countries) plus the European Union, which funds research into complex biological systems. Among foundations, the Howard Hughes Medical Institute, the Wellcome Trust, and the American Heart Association are among the fifty top research sponsors of synthetic biology papers, while the Welch Gates, and Packard foundations are just outside the top 50. The Council of Scientific and Industrial Research (India) and the Russia Foundation for Basic Research are the only other emerging economy sponsors among the top 50 research sponsors, with each at sponsorships levels that are about one-twentieth of the number of papers acknowledged by Chinese research funders.

Among the leading research sponsors of synthetic biology papers, there are noticeable differences in macro-disciplines of focus. For example, 44% of papers with acknowledgements to MEXT are in “chemistry and biochemical research” methods, while about 40% respectively of synthetic biology papers with acknowledgements to the UK EPSRC and the US DOD are also in this macro-discipline. For CIHR, NIH and DARPA, there is a relatively greater focus on “biochemistry and molecular and cell biology”, with over 30% of the synthetic biology papers funded by each of these sponsors appearing in this macro-discipline. In China, “biotechnology” is the prioritized macro-discipline for the 863 and 973 Programs, acknowledged by 54 and 41% respectively of synthetic biology papers supported by these two funding sources.

In addition to influencing research themes, research sponsorship also has a relationship on the likelihood of research impacts. In particular, synthetic biology papers that acknowledge funding sources are more likely to be cited than papers which do not report any funding information. Synthetic biology papers published between 2009 and 2015 garnered an average of 16.1 citations per paper (as of May 2016); those with funding acknowledgements (more than four-fifths of the papers) averaged 18.0 citations per paper compared with 8.4 citations for those without funding acknowledgements. This is consistent with findings previously reported that funded papers garner higher citation counts than papers which do not reveal any funding for reasons which can include higher motivation and access to resources for funded researchers and the role of review and selection processes in filtering out weaker research and rewarding promising topics, as well as potential reputational biases associated with funding awards and paper citations (Wang and Shapira 2015). We note other interaction effects. For example, we observe significant field differences in citation propensities. It is well established that citations to research journals vary significantly by fields, with research in fields such as cell biology, multidisciplinary chemistry, nanoscience, and multidisciplinary science more likely to attract higher citations (on average) than research in mathematics and statistics, branches of engineering, computer science, and social science (Thomson Reuters 2016). These field differences are influenced by factors that include the size of the field, numbers of authors, and field-specific citation practices, while within fields only a minority of papers and journals become very highly-cited (Garfield 1996; Aksnes 2003; Ioannidis 2006). We observe such variations in our data set. For synthetic biology papers published between 2009 and 2015, the mean citation rate per paper for research in the WoS macro-discipline of “biochemistry and molecular and cell biology” was 17.8, with papers in “chemistry and biochemical research” and in “biotechnology” each attracting around 13 cites per paper on average. Synthetic biology papers in “genetics; computational biology” and in “computer science and engineering” respectively averaged 10.6 and 6.3 citations per paper. Most noticeably, synthetic biology papers in the macro-discipline that covers “multidisciplinary sciences” garnered 33.4 cites per paper on average. Indeed, 28 of the top 50 cited synthetic biology papers published between 2009 and 2015 were in “multidisciplinary sciences” including 11 papers in *Nature* and 10 papers in *Science*—two journals with among the highest journal impact factors of all publications in the WoS (Thomson Reuters 2016). Five other macro-disciplines were represented among this group of 50 most cited synthetic biology papers: “biochemistry; molecular and cell biology” (with 14 highly-cited papers), “biotechnology” (5 highly-cited papers), “chemistry and biochemical research methods” (3 highly-cited papers), and “biology and ecology” and “material sciences” (each with one highly-cited paper). (Total adds to more than 50 due as two journals are classed in more than one macro-disciplinary category.)

Among the sponsors of research, we also observe differences in citations accrued by synthetic biology papers published between 2009 and 2015 and which acknowledge specific sponsors (Fig. 4). It should be noted that papers published in this time period could have been sponsored prior to the start of the time period, citations tend to increase over time (meaning that papers published towards the start of the period have more time to accrue citations), and that papers typically acknowledge more than one funding sponsor. Additionally, variances in citation propensities by field (as noted above, given our results on how research sponsors vary in their fields of supported research), and in citation practices by and of authors from different countries (Tang et al. 2015; Albarrán et al. 2015), should be kept in mind. NIH and NSF—the two research sponsors with the greatest number of synthetic biology papers published between 2009 and 2015 that acknowledge their support—also have accrued a disproportionately high number of citations (about 20,500 and 16,000 respectively). The papers associated with these two sponsors have relatively high average citation rates—about 29 and 26 cites per paper respectively for those sponsored by NIH and NSF. Synthetic biology papers which acknowledge DOD, CIHR, and DOE also attract a comparably high number of average citations per paper. Synthetic biology papers acknowledging the US ONR attract the greatest number of average citations per paper (more than 40). Among the top 50 most cited synthetic biology papers, 30 acknowledge NIH while 26 acknowledge NSF. (Again, totals exceed 50 as papers acknowledge multiple research sponsors). Just over 4% of all synthetic biology papers sponsored by NIH and NSF (and published between 2009 and 2015) are included in the top 50 most cited group. ONR sponsorship is associated with the third highest number of top cited papers, although it is 13th in total number of papers sponsored. Over 8% of all ONR sponsored papers are included in the top 50 most cited group. All papers that acknowledge ONR also acknowledge other sponsors, most commonly NIH and NSF. Lower average citation levels per paper are seen in synthetic biology papers sponsored by China’s 873 and 973 programs, and by the Korean National Research Foundation. Neither of the two Chinese governmental programs had any sponsored papers in the top fifty most cited papers, although three papers sponsored by NSFC of China were in this top group although none were in *Nature* or *Science*. Synthetic biology papers sponsored by European funders generally had average citation impacts at levels lower than for US sponsors, although there was some placement of European-sponsored synthetic biology papers in high impact multidisciplinary and field journals.

## Discussion and conclusions

This paper has put forward a systematic approach to bibliometrically defining the research domain of synthetic biology. We constructed a keyword based search strategy using keyword co-occurrence analysis, combined this keyword-based search strategy with a targeted synthetic biology journal search, and undertook iterative refinement processes. We identified and cleaned a dataset of synthetic biology publications published between 2000 and 2015. Our approach, when compared with other narrower and broader synthetic biology search strategies, resulted in reasonably robust balances of precision and recall.

The search strategy described in this paper is positioned as a public tool that is available for use and refinement by the research community. Researchers, technology managers, research sponsors, policy analysts and others who seek information on scientific and technological development in the synthetic biology field can use the search strategy. A database of synthetic biology papers can be generated by applying the search strategy (comprising the collated key word terms in Table 3 plus the journals listed in Table 4) to the Web of Science. The search strategy can be applied to other databases of publications (such as PubMed or Scopus) and to patents, bearing in mind variations in how search algorithms and key terms are entered into these databases. The limited search features of Google Scholar makes it less amenable to complex bibliometric searches.

Given the continued pace of growth and development of research in synthetic biology, the search approach and analysis contained in the paper inevitably represents an *ex post* view. This retrospective limitation should be kept in mind when using and interpreting the results. Our initial set of candidate keywords was garnered from papers captured by the MeSH term for synthetic biology in PubMed for records published from 2011 to 2014; and, after the further steps detailed earlier in this work, we applied our approach to papers published in the period from 2000 to 2015. Over time, it is likely that scientific advances will generate new synthetic biology terms that were not evident or captured when we developed the search approach. As an update option, users can add new synthetic biology journals or curated collections to the journal search list. At a future point, it could also be useful to revisit the keywords captured by the MeSH synthetic biology classification and in papers included in synthetic biology journals or curated collections.

In the paper, we applied our approach to track developments in synthetic biology research and publication, including analyses of the key scientific disciplines and knowledge sources contributing to this emerging field, national outputs and international collaborations, the distribution of synthetic biology on the map of science, and its profile of research sponsorship. These are selected examples of the multiple-kinds of analyses that can be undertaken with the approach. There are many opportunities to undertake further analyses, with controls and drawing on added data sources, to probe issues related to research and governance in the synthetic biology domain. As noted, there are also opportunities to further refine our search strategy, especially as synthetic biology and its research community evolves, and to apply the search strategy principles to develop bibliometric search approaches in related as well as non-related emerging technology domains.

The analysis in this paper details the rapid growth and international spread of research in synthetic biology in recent years, with an expansion of research nodes in the US and Canada, Europe, and East Asia. We show that diverse research disciplines are contributing to the global growth of synthetic biology research outputs in recent years. These include clusters in biochemistry, molecular and cell biology, and chemistry and biochemical research methods, but also engagement from multidisciplinary sciences, genetics and computational biology, microbiology, and energy and fuels. Engineering disciplines and approaches are distributed across in multiple sub-fields in the development of synthetic biology. Most significantly, the range of research fields involved in synthetic biology implies that it is emerging as an assembly of platform technologies that is likely to lead to multiple applications in biochemical, medical and health, energy, materials, agricultural, and other markets. We also observe small streams of research outputs that probe ethical, legal, societal, and governance aspects of synthetic biology, although it is uncertain whether this work is yet of sufficient scale, embeddedness, and impact in terms of its contribution to responsible research and innovation in synthetic biology.

Inherently, further questions arise from the analyses reported in the paper. For example, we highlight the key roles played by a relatively concentrated set of about 20 research sponsors in funding the growth and trajectories of synthetic biology. It would be appropriate, in follow-on research, to probe in detail the policies and procedures adopted by this dominant grouping of research sponsors, who are located in North America, Europe, and Asia, and how these will impact the further development of the synthetic biology domain and its governance. At a program evaluation level, we can see cases where research sponsorship (with significant funding levels) has been explicitly targeted to stimulate synthetic biology research, including large-scale synthetic biology research centers in the US, and synthetic biology networks and research centers in the UK and elsewhere. In other cases synthetic biology research has been sponsored through existing funding arrangements. It seems that the clustering of synthetic biology research in dedicated centers has broadly contributed to growth and recognition of the domain, but many questions have yet to be addressed about the performance of individual centers, their levels of interdisciplinary, and their approaches to responsible governance, training, and commercialization.

Our search approach operationalizes a feasible and replicable bibliometric method to capture research outputs in the synthetic biology domain. We acknowledge the various limitations of the approach. These include the comparative limitations of the Web of Science (Falagas et al. 2008; Leydesdorff et al. 2016) including weaknesses in terms of the scope of publications and disciplines that are indexed and incompleteness in some fields (for example, funding acknowledgements). We also recognize the imperfections in using publications and citations as measures of research performance, quality, and impact. Importantly, while we appreciate, and attempt to address, the ambiguities that currently exist in the understanding of what synthetic biology is, we cannot resolve all uncertainties about the delineation of synthetic biology. There are underlying differences in terms of expert and governance interpretations as to what constitutes the field, and advancement in the field itself, including convergence with other technologies and disciplines, which will further confound definitional approaches. Our operationalization of a bibliometric definition of synthetic biology is situated within this disputed terrain. Although we have carefully deliberated upon and tested our approach, probably our search strategy does not capture every relevant keyword that can be representative of synthetic biology research (although it is always important to check whether other included terms are already capturing anything that is felt to be missing), and some may judge that certain terms are too expansive. Moreover, with further iterations of the search strategy, additional exclusion terms might be appropriate. It will thus be useful to continue to probe our approach, to test and refine it through further studies, and also to explore how other bibliometric approaches, including those that use enhanced machine learning algorithms to discern what is (and is not) in the domain, compare with the keyword (plus key journal) synthetic biology search strategy that we have put forward in this paper.
